# Supplementary material for: Colorectal Cancer Stage-Specific Fecal Bacterial Community Fingerprinting of the Taiwanese Population and Underpinning of Potential Taxonomic Biomarkers
Source: Microorganisms. 2021 Jul 21;9(8):1548. doi: 10.3390/microorganisms9081548 (PMC8401100; doi:10.3390/microorganisms9081548)
Supplement: Supplementary file 1 [file microorganisms-09-01548-s001.zip › microorganisms-1278925-supplementary.pdf]

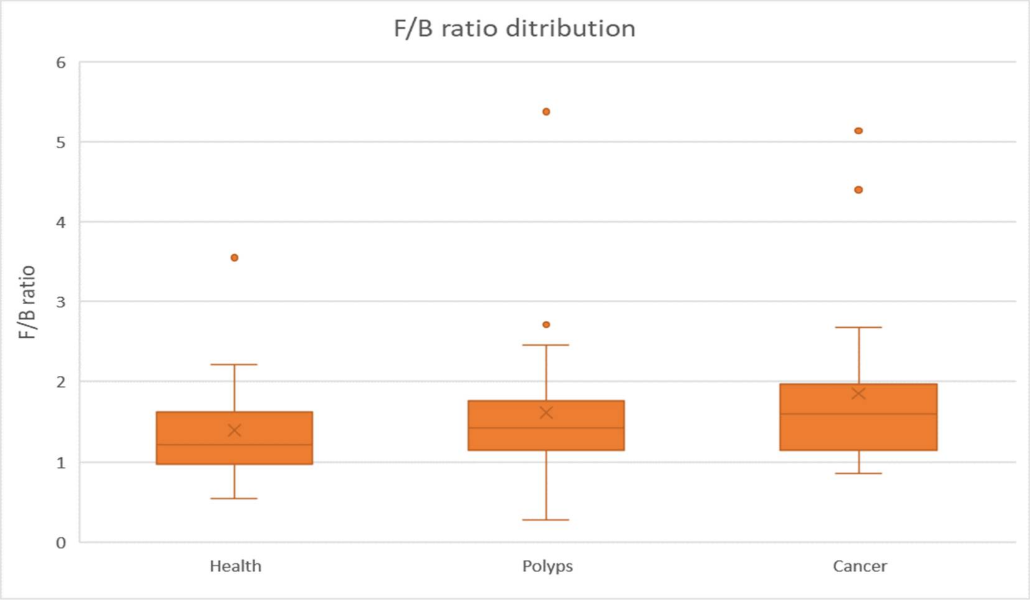

Supplementary Figure S1. Ratio of Firmicutes to Bacteroides in experimental groups.

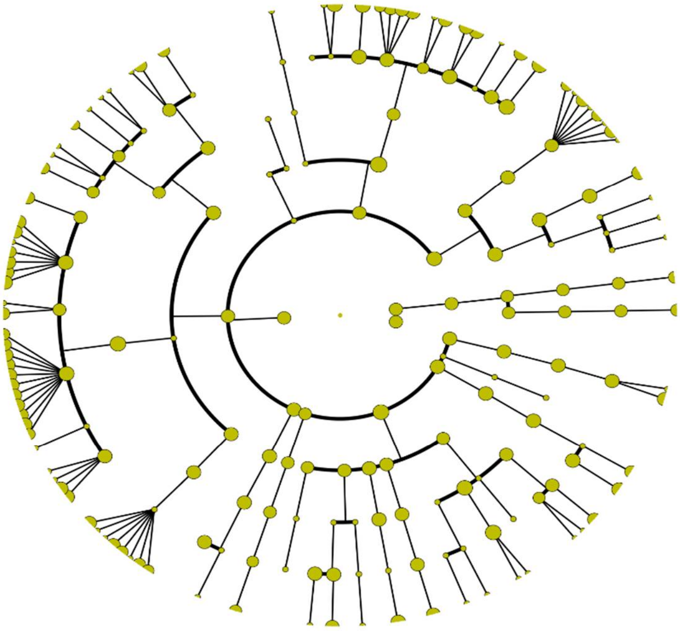

Supplementary Figure S2. Cladogram plot of LEfSe analysis among the experimental groups

Supplementary Table S1. Correlation analysis between the predicted functions and bacterial taxa at the genus level.

ID	Biosynthesis of other secondary metabolites	Cell growth and death	Glycan biosynthesis and metabolism	Lipid metabolism	Metabolism of cofactors and vitamins	Metabolism of other amino acids	Metabolism of terpenoids and polyketides	Xenobiotics biodegradation and metabolism	Cancer: overview	Cancer: specific types	Digestive system	Signal transduction	Substance dependence	Transcription
Actinomyces	.260*	.260*	0.208	0.242	0.085	0.243	0.249	.265*	0.232	0.243	0.221	0.159	0.249	0.092
Atopobium	.281*	.276*	0.189	0.257	0.021	0.225	.272*	.281*	0.221	.268*	0.207	0.063	.283*	0.094
Collinsella	0.239	0.230	0.179	0.226	0.108	0.199	0.243	.274*	0.233	0.239	0.224	0.085	.283*	0.175
Eggerthella	.317*	.282*	.264*	.345**	0.004	.275*	.285*	.355**	.275*	.305*	0.215	0.018	.300*	0.076
Prevotella	-0.227	-0.168	-.308*	-.274*	-0.154	-0.229	-0.199	-0.228	-0.246	-0.178	-0.206	-0.154	-0.214	-0.254
Enterococcus	-0.050	-0.022	0.141	-0.003	0.216	0.099	-0.068	-0.003	0.122	-0.026	0.139	.267*	-0.074	0.055
Olsenella	-0.053	-0.024	0.134	-0.008	0.218	0.094	-0.071	-0.016	0.114	-0.048	0.147	.269*	-0.106	0.166
Slackia	0.225	0.230	0.181	0.226	0.061	0.208	0.225	0.225	0.214	.260*	0.202	0.089	0.181	0.083
Bacteroides	.283*	.326*	.449**	.317*	.379**	.348**	0.246	.283*	.337**	.322*	.388**	.348**	0.199	0.177
Odoribacter	-.264*	-0.241	-0.053	-.268*	0.180	-0.203	-.285*	-.291*	-0.188	-0.240	-0.133	0.095	-.261*	-.266*
Paraprevotella	-0.015	-0.007	0.111	0.007	.269*	0.034	-0.035	-0.007	0.099	0.017	0.096	0.209	-0.066	-0.047
Elizabethkingia	-0.249	-0.248	-0.170	-0.238	0.147	-0.147	-.271*	-0.226	-0.142	-0.220	-0.175	0.074	-0.216	-0.247
Bacillus	0.238	0.234	.273*	0.256	0.084	0.221	0.224	0.246	0.234	0.219	0.187	0.144	0.233	0.155
Streptococcus	.281*	.274*	0.239	.282*	0.089	.286*	0.252	.297*	.269*	0.195	0.232	0.143	.285*	0.207
Clostridium	.383**	.393**	0.231	.359**	0.026	.267*	.391**	.358**	.259*	.339**	.270*	0.047	.368**	.280*
Dorea	.431**	.416**	.291*	.445**	0.079	.354**	.427**	.428**	.397**	.331*	.335*	0.136	.443**	.414**
Ruminococcus	.340**	.376**	.284*	.372**	0.093	.312*	.336*	.361**	.341**	.332*	.311*	0.129	.298*	0.194
Peptostreptococcus	0.196	0.205	0.162	0.204	-0.027	0.102	0.196	0.201	0.149	.284*	0.121	-0.003	0.155	0.115
Anaerotruncus	0.251	0.252	0.184	0.251	0.012	0.161	0.243	0.231	0.181	.263*	0.165	0.041	0.216	0.197
Sporobacter	0.203	0.238	0.088	0.198	-0.132	0.134	0.199	0.221	0.091	.319*	0.075	-0.077	0.206	0.067

Holdemania	0.179	0.150	0.138	0.169	0.044	0.176	0.173	0.177	0.166	0.110	0.150	0.133	0.140	.317*
Desulfovibrio	-.273*	-.338**	-.326*	-.290*	-0.128	-.292*	-0.247	-.294*	-.301*	-.274*	-.317*	-0.199	-.286*	-0.241
Aeromonas	0.178	0.161	0.127	0.178	0.070	0.172	0.184	0.178	0.173	0.132	0.109	0.161	0.231	.282*
Succinatimonas	-0.127	-0.127	-0.244	-0.154	-.263*	-0.177	-0.041	-0.132	-0.210	-0.086	-0.211	-.282*	-0.046	-0.108
Pseudomonas	-0.010	0.027	0.140	0.008	.295*	0.071	-0.019	-0.011	0.103	-0.001	0.125	.263*	-0.015	0.131
Akkermansia	0.219	0.225	0.233	0.233	0.101	0.243	0.213	0.230	0.234	.267*	0.216	0.219	0.154	0.225

** Correlation is significant at the 0.01 level (2-tailed); * Correlation is significant at the 0.05 level (2-tailed).

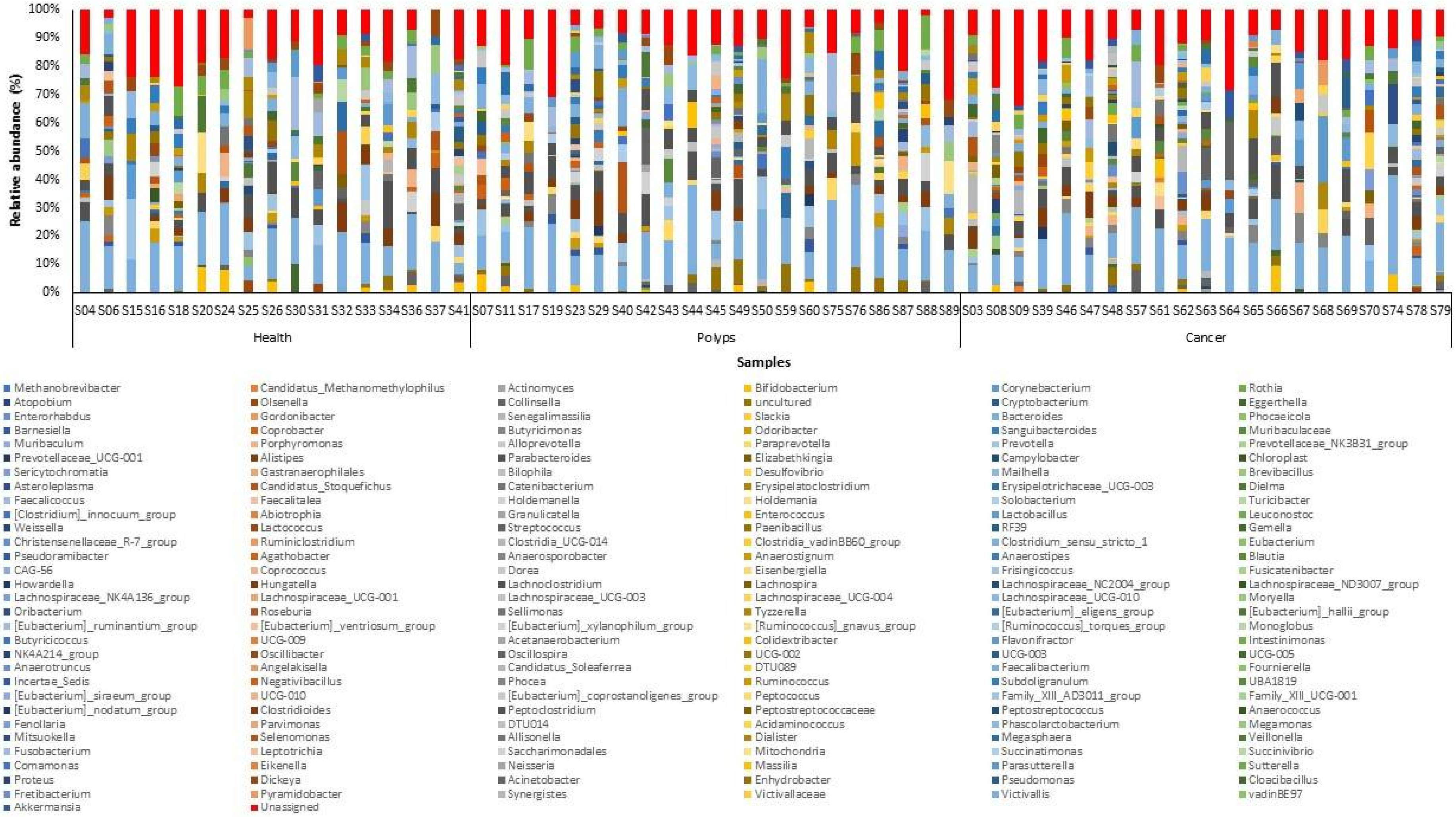

Supplementary Figure S3. 16S rRNA amplicon sequencing based relative abundance of microbial diversity at genus level among the experimental groups using SILVA database.
